# Functional and Oncological Outcome of Patients with Distal Femoral Osteosarcoma Managed by Limb Salvage Using Modular Endoprosthesis

**DOI:** 10.1245/s10434-023-13679-x

**Published:** 2023-06-05

**Authors:** Wael Mohamed Safwat Sadek, Walid Atef Ebeid, Ahmed El Ghoneimy, Emad Ebeid, Wessam Gamal Abou Senna

**Affiliations:** 1 Department of Orthopaedics and Traumatology, Cairo University, Cairo, Egypt; 2 Department of Paediatric Oncology and Haematology, National Cancer Institute, Cairo University, Cairo, Egypt

**Keywords:** Modular endoprosthesis, Distal femur osteosarcoma, Limb salvage, Complications

## Abstract

**Background:**

The aim of the study was to assess the functional and oncological outcomes of patients with distal femoral osteosarcoma managed by limb salvage using modular endoprosthesis as well as to assess related complications.

**Patients and Methods:**

A total of 82 patients were included in our study. Functional outcome was assessed using MSTS score and knee range of motion. Oncological outcome was assessed regarding local recurrence, chest metastasis, and patient survivorship. Complications were classified according to Henderson et al.

**Results:**

The mean MSTS score was 26.21 (87.4%) (range 8–30 points) with 70.7% of patients having more than 90° of flexion. The incidence of local recurrence was 3.7% (3 patients), while the incidence of chest metastasis was 14.6% (12 patients). Aseptic loosening (type 2 failure) was the commonest complication (19.5%), followed by infection (15.9%). The 5- and 10-year survivorships of the limb were 98.8%, while the 5- and 10-year survivorships of the prosthesis were 67.7% and 52.4%, respectively.

**Conclusion:**

This study showed that patients with osteosarcoma distal femur who are treated by chemotherapy and limb salvage have an excellent long-term prognosis in terms of patient as well as limb survivorship. The use of modular endoprosthesis in these patients offer an acceptable function, with two-thirds of the patients retaining their prosthesis after 5 years and more than half retaining them after 10 years.

Distal femur is the commonest site for osteosarcoma. The introduction of chemotherapy and better imaging by MRI has improved the survivorship of these patients and shifted the treatment toward limb salvage.^1–4^ Several options are available for reconstruction after distal femoral resection. Many factors may alter the surgeon’s reconstructive choice. Modular endoprosthesis presumably can reconstruct large osteoarticular defects and allow for the immediate return of function. However, they are expensive and are associated with considerable complications. Moreover, they do not offer a permanent reconstruction.

The aim of this study was to answer the following questions:

1. What are the functional and oncological outcomes of osteosarcoma patients managed by limb salvage using modular endoprosthesis?
2. What are the complications associated with modular endoprosthesis?
3. What is the rate of limb salvage and prosthesis preservation in those patients?

## Patients and Methods

This retrospective audit of a prospective single-center database was conducted after approval of the ethical committee of our institute. Informed consent was obtained from all patients and this study followed the principles of the Declaration of Helsinki. A total of 82 patients with distal femoral osteosarcoma were included in the study; with 41 male patients and 41 female patients and a mean age of 20.13 ± 8.72 years (range 12–57 years). They were all managed by wide intraarticular resection and reconstruction with a modular endoprosthesis.

Our exclusion criteria were multifocal, secondary, and recurrent osteosarcoma and extraarticular or intercalary resection, as well as patients who were metastatic at presentation or patients who were managed by expandable prosthesis or total femur replacement.

Knee range of motion and isometric exercises as well as partial weight bearing (for 3 weeks postoperatively to allow for muscle strengthening and rehabilitation) were allowed immediately postoperative for patients managed by cemented prosthesis. Weight bearing was delayed for 4–6 weeks in patients managed by cementless prosthesis or in patients in which intraoperative fractures occurred.

Follow-up included radiological, clinical, and functional assessment at 3 weeks, then every 6–8 weeks for year 1, then every 3 months for years 2–3, then every 6 months for years 4–5, then annually. Complications and failures were classified according to Henderson et al.: type 1, soft tissue failures (flap insufficiency, skin necrosis, and stiffness/contracture of the reconstructed joint were considered as soft tissue failure); type 2, aseptic loosening failures; type 3, structural failures, that is, fractures of prosthetic components; type 4, infection; and type 5, tumor prolapse/progression.^5^

**Figure 1:**
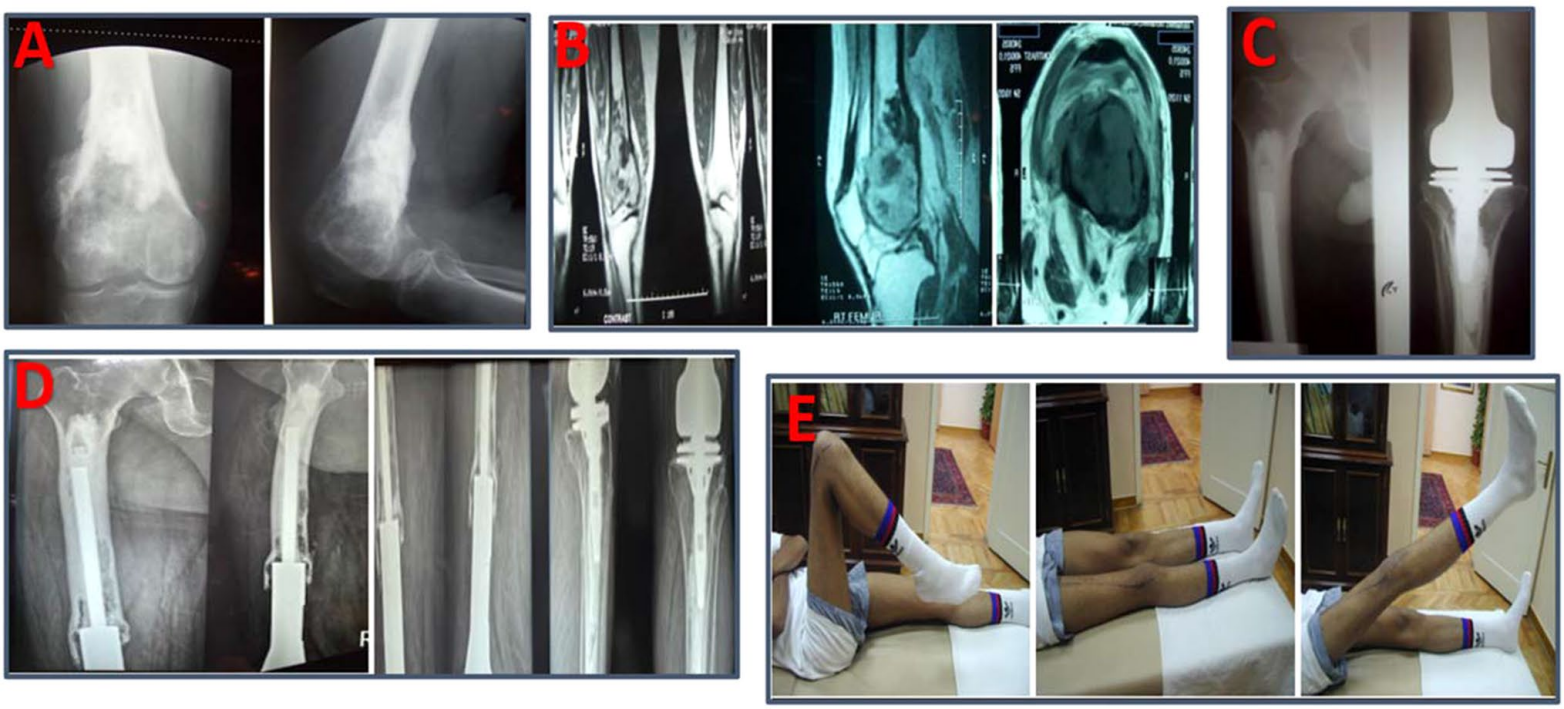
57 years of male with right distal femoral osteosarcoma and CT chest was free. **A** Pre-operative X-rays. **B** Pre-operative MRI. **C** Post-operative. **D** 11 years follow-up X-rays. **E** Final range of motion with MSTS score 30

Functional outcome was assessed at every follow-up visit using the Musculoskeletal Tumor Society system (MSTS) score and by measuring the range of motion. Oncological outcome (local recurrence and chest metastases) was assessed by clinical and radiological evaluation. Functional and oncological outcomes were recorded according to the last follow-up visit or until amputation/death of the patient (Fig. 1).

Data were coded and entered using the Statistical Package for the Social Sciences (SPSS) version 26 (IBM Corp., Armonk, NY, USA). Data were summarized using mean, standard deviation, and minimum and maximum in quantitative data, as well as frequency (count) and relative frequency (percentage) for categorical data. For comparing categorical data, the chi-squared (*χ*^*2*^) test was performed. Exact test was used instead when the expected frequency was less than 5.^6,7^ Survival curves were plotted by the Kaplan–Meier method. *p*-Values < 0.05 were considered as statistically significant.

## Results

The patients were managed between 2000 and 2019. Mean follow-up period was 73.39 months (range 12–230 months). A total of 16 patients (19.5%) presented with pathological fractures at time of diagnosis. All patients received neoadjuvant and adjuvant chemotherapy according to the European and American Osteosarcoma Study (EURAMOS) protocol, and surgeries were performed by surgeons at the same institute.

Routine staging, including plain x-ray whole femur and knee, MRI whole bone with contrast, bone scan, computed tomography (CT) chest, and finally biopsy were carried out for all patients. The mean operative time was 3.55 h (range 2–6 h). The mean resected length of bone was 16.70 cm (range 10–26 cm). Medial approach was used in 48 patients (58.5%), lateral approach in 33 patients (40.2%), and double approach in one patient. The resected specimen was sent for histopathological examination to confirm diagnosis and assess the margins as well as the tumor necrosis.

A total of 60 patients were managed by cemented prosthesis (73.2%), while only 22 were managed by cementless type (26.8%). This was solely due to availability, as early in this study, the only available prostheses were cemented. All the prostheses used in this study were rotating-hinge prostheses, except for two that were fixed-hinge.

### Functional Outcome

MSTS scores of 27–30 were classified as excellent (50 patients), 23–26 as good (21 patients), 16–22 as fair (6 patients), and 15 or less as poor (5 patients). The mean score was 26.21 points (87.4%) (range 8–30 points) (Table 1).

**Table 1:** Functional outcome

	Mean	SD	Minimum	Maximum
MSTS score	26.21	4.68	8.00	30.00
Pain	4.76	0.71	2.00	5.00
Function	3.83	0.90	2.00	5.00
Emotional	4.61	0.93	1.00	5.00
Support	4.61	1.02	1.00	5.00
Walking ability	4.15	1.01	0.00	5.00
Gait	4.26	1.08	1.00	5.00

		Count	%
ROM	0–40	13	15.9
	40–90	11	13.4
	> 90	58	70.7
Flexion deformity		8	9.8
Stiff knee		7	8.5
Extension lag		7	8.5

MSTS score was statistically significantly affected by infection and aseptic loosening (*p* ˂ 0.05), while the range of motion was significantly affected by infection only (*p* = 0.034). A multivariate linear regression was done to detect independent predictors of MSTS score, and it showed that the only significant predictor of MSTS score was infection being in negative relation to the score (Table 2). Other factors, such as age, sex, size, surgical approach, pathological fracture on presentation, and cemented or cementless prosthesis, as well as extension lag, had no impact on the functional outcome.

**Table 2:** Multivariate linear regression of MSTS score

Model		Unstandardized coefficients		Standardized coefficients	*t*	*p*-Value	95.0% confidence interval for B	
		*B*	SE	Beta			Lower bound	Upper bound
MSTS score	(Constant)	26.505	3.231		8.204	< 0.001	20.063	32.946
	Follow-up period in years	0.061	0.138	0.055	0.439	0.662	−0.215	0.336
	Age	0.020	0.062	0.037	0.317	0.752	−0.104	0.144
	No. of muscles resected	−0.557	0.768	−0.084	−0.725	0.471	−2.089	0.975
	Length resected	0.061	0.159	0.044	0.383	0.703	−0.256	0.378
	Pathological fracture	0.594	1.400	0.051	0.424	0.673	−2.198	3.386
	Cemented or not	−0.353	1.281	−0.034	−0.275	0.784	−2.907	2.202
	Aseptic loosening	−1.883	1.445	−0.160	−1.303	0.197	−4.765	0.999
	Infection	−3.354	1.427	−0.263	−2.350	0.022	−6.200	−0.508
	Fracture	−0.639	1.904	−0.043	−0.336	0.738	−4.435	3.157
	Broken prosthesis	−2.932	2.191	−0.164	−1.338	0.185	−7.300	1.436

### Oncological Outcome

The incidence of local recurrence was 3.7% (three patients); two of them were managed by wide resection and one was managed by hip disarticulation. The incidence of chest metastasis was 14.6% (12 patients); 6 patients were managed by metastatectomy, while the other 6 were managed by chemotherapy only. The average time of diagnosis of metastasis was 32.6 months (range 2–156 months).

### Complications

**Figure 2:**
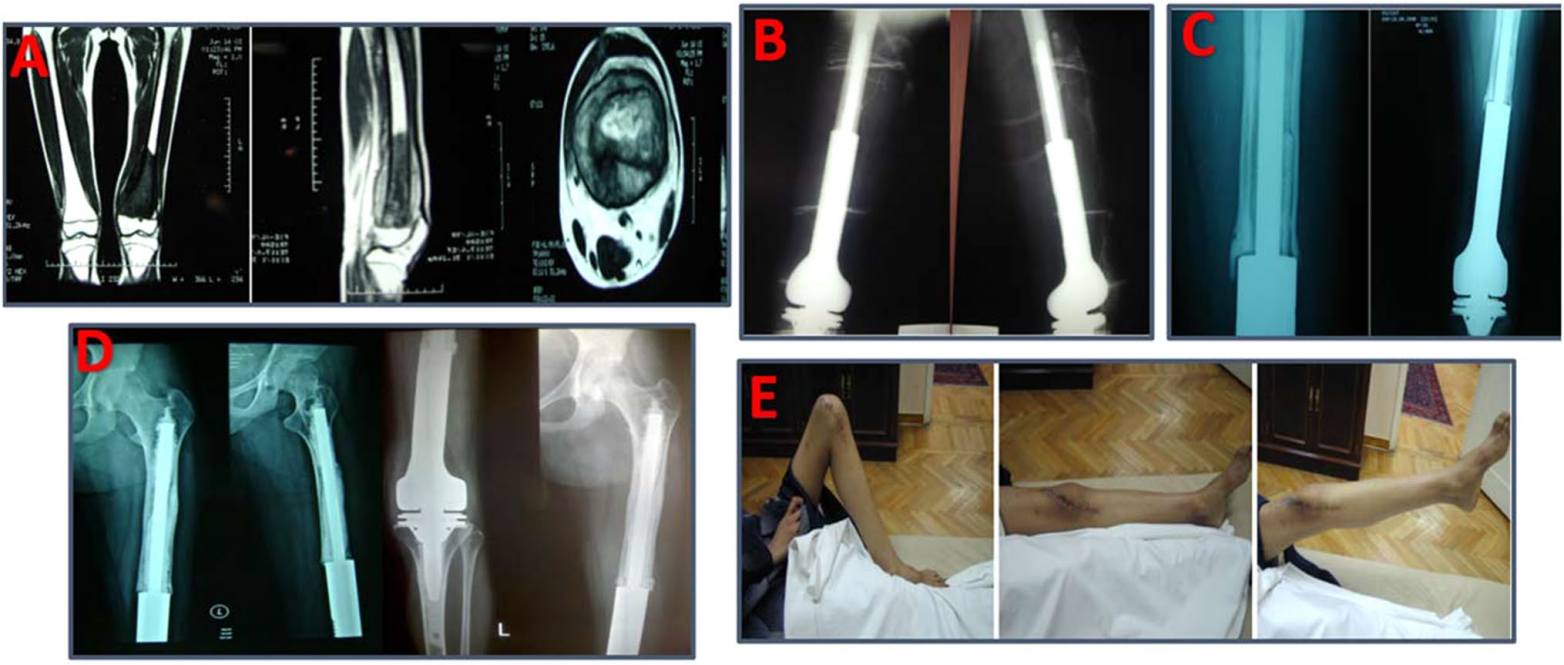
15 years old female with left distal femoral osteosarcoma and CT chest was free. **A** Pre-operative MRI. **B** Post-operative X-ray. **C** 6 years follow-up showing loosening of the femoral stem. **D** Post-revision X-rays. **E** Final range of motion with MSTS score of 24 (s years after the revision)

A total of 47 failures occurred in 36 implants. Seven patients experienced more than one type of failure. Aseptic loosening (type 2 failure) was the most common complication (19.5%) (Fig. 2). The incidence of aseptic loosening was higher in cemented prosthesis but not statistically significant (*p* = 0.057). The average time for detecting loosening was 4.6 years (range 1–14 years). All were managed by single-stage revision (15 patients), except one who was managed by total femur replacement.

The incidence of infection (type 4 failure) was 15.9% (13 patients). Infection was considered to be “early” (type 4A) if infection developed within 2 years after implantation of the prosthesis (9 patients) and “late” (type 4B) if infection developed after 2 years (4 patients). The incidence of broken prosthesis (type 3A) and periprosthetic fracture (type 3B) was 7.3% (6 patients) and 11% (9 patients), respectively.

### Prosthesis, Limb, and Patient Status

A total of 31 prostheses (37.8%) were removed due to different causes (Table 3). Six patients (7.3%) died and two underwent amputation; one due to persistent infection and one due to extensive local recurrence.

**Table 3:** Prosthesis status

Prosthesis status	Removed	31	37.8%
	Not	51	62.2%
Indication of removal	Aseptic loosening	16	
	Infection	11	
	Broken prosthesis	6	
	Periprosthetic fracture	1	
	Infection	1	
	More than one cause	7	
Timing of removal	During the first year	6	19.3%
	1–5 years postoperative	18	58.1%
	6–10 years postoperative	4	12.9%
	More than 10 years postoperative	3	9.7%
Management	Single-stage revision	21	67.9%
	Spacer only	4	12.9%
	Two-stage revision	2	6.4%
	Amputation	2	6.4%
	Spacer then VF	1	3.2%
	Total femur	1	3.2%

### Prosthesis, Limb, and Patient 5- and 10-Year Survivorship

**Fig. 3:**
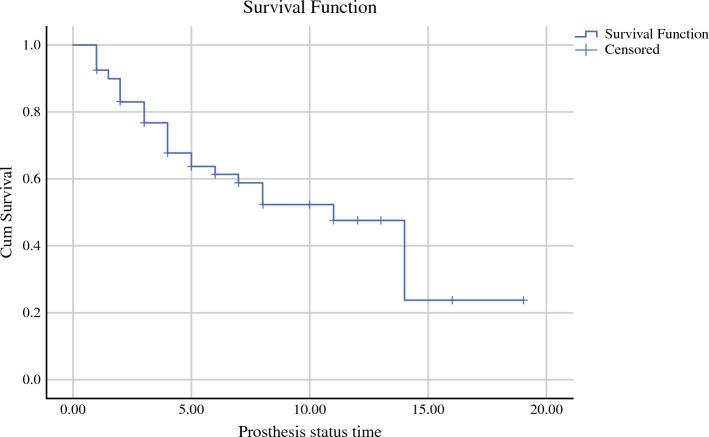
Kaplan-Meier curve for prosthesis survivorship

**Fig. 4:**
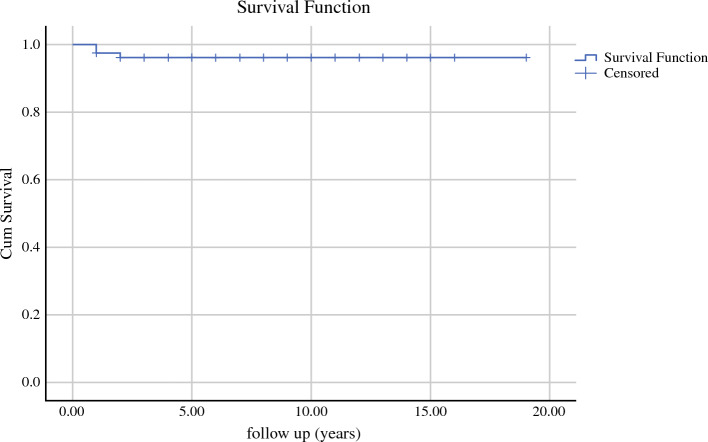
Kaplan-Meier curve for recurrence-free survivorship

Using Kaplan–Meier curves, the 5- and 10-year rates of prosthesis preservation were 67.7% and 52.4%, respectively (Fig. 3). The 5- and 10-year rates of limb salvage were 98.8%, while the 5- and 10-year survivorships of the patients were 95.6% and 91.4%, respectively (Fig. 4). Multivariate analysis and Cox regression analysis were done to assess the factors that increase the risk for prosthesis removal (Table 4).

**Table 4:** Multivariate analysis and cox regression for prosthesis preservation

		Prosthesis preservation				*p*-Value
		Removed		Not		
		Count	%	Count	%	
Age groups	12–18 years	12	38.7%	33	64.7%	0.063
	19–40 years	17	54.8%	16	31.4%	
	Older than 40 years	2	6.5%	2	3.9%	
Sex	Male	17	54.8%	24	47.1%	0.494
	Female	14	45.2%	27	52.9%	
Resection length	15 cm or less	6	19.4%	21	41.2%	0.111
	16–20	21	67.7%	24	47.1%	
	More than 20	4	12.9%	6	11.8%	
No. of muscles resected	0	2	6.5%	7	13.7%	0.217
	1	12	38.7%	25	49.0%	
	2	17	54.8%	17	33.3%	
	3	0	0.0%	2	3.9%	
Approach	Lateral	14	45.2%	19	37.3%	0.778
	Medial	17	54.8%	31	60.8%	
	Medial and lateral	0	0.0%	1	2.0%	
Cemented or not	Yes	27	87.1%	33	64.7%	0.026
	No	4	12.9%	18	35.3%	
Aseptic loosening	Yes	16	51.6%	0	0.0%	< 0.001
	No	15	48.4%	51	100.0%	
Infection	Yes	12	38.7%	1	2.0%	< 0.001
	No	19	61.3%	50	98.0%	
Fracture	Yes	5	16.1%	4	7.8%	0.288
	No	26	83.9%	47	92.2%	
Broken prosthesis	Yes	6	19.4%	0	0.0%	0.002
	No	25	80.6%	51	100.0%	

	*p*-Value	HR	95.0% CI	
			Lower	Upper
Cemented or not	0.323	0.557	0.175	1.777
Aseptic loosening (type 2)	< 0.001	6.451	2.698	15.427
Broken prosthesis (type 3)	< 0.001	7.816	2.571	23.768
Infection (type 4)	< 0.001	4.616	2.083	10.226

## Discussion

Decades ago, patients with osteosarcoma were only demanding better survivorship. Nowadays patients not only seek limb preservation, but they also expect a good functioning limb. There are different reconstructive options for limb salvage after wide resection of distal femoral osteosarcoma. Modular endoprosthesis is one of the favorable options, as it allows for the reconstruction of large defects and for faster return to activity.

Regarding functional outcome, MSTS scores were good or excellent in 86.6% of patients, with an average score of 26.21 (87.4%). In total, 70% of our patients achieved more than 90° of flexion, with mean range of flexion 104.6° (range 20–130°). These results were comparable or slightly better than most of the literature.^8–16^ Most of the studies included benign tumors as well as malignant tumors other than osteosarcoma. Moreover, some of these studies included proximal tibial endoprosthesis as well.

The second aim of this study was to evaluate the oncological outcome of the patients. Local recurrence occurred in three patients (3.7%). Two were managed by wide resection and one underwent hip disarticulation. Incidence of local recurrence was not significantly affected by age, sex, resection length, or pathological fracture. Other long-term follow-up studies have reported rates between 2.9% and 9%.^9,10,17–20^

A total of 12 patients developed chest metastasis in our study (14.6%). Six patients died; five developed metastasis and one did not. The five patients who developed chest metastasis and died were managed by either medical treatment (four patients) or metastatectomy (one patient). The 5- and 10-year survivorships of the patients were 95.6% and 91.4%, respectively. This is higher than most of the literature (60–80%),^9,11,21–23^ and may be attributed to the patient selection criteria used in this study. Patient survivorship was affected significantly by chest metastasis but not by local recurrence, as their numbers were low.

The third question that was tackled by our study was the incidence of complications associated with this procedure. Mechanical failure occurred in 26 patients (31.7%), whereas non-mechanical failure occurred in 16 patients (19.5%). The failure rates of modular prosthesis in the literature ranged from 21.6% to 61%,^16,24–26^ however, more recent literature has shown the incidence of failures ranging from 26% to 35%.^9,13,15,17^ The incidence of failure in our study was slightly higher than most of the recent literature. This may be because most of those studies included benign tumors as well as metastasis, and this would affect the incidence of complications, as those patients did not receive adjuvant treatment as chemotherapy and did not require aggressive dissection and resection of large number of muscles in most of the cases.

Aseptic loosening (type 2 failure) was the most common type of failure in our study (16 cases, 19.5%). This incidence is similar to the literature, as most of the authors report it as the most common type of failure in long-term follow-up with a range of 3–29.9%.^15,16,24,27^ The incidence of aseptic loosening was much lower in cementless prostheses; however, the difference was not statistically significant. This may be due to the small number of cementless prostheses in this study. Some authors believe cemented implants are associated with lower incidence of aseptic loosening;^28,29^ they thought that osteointegration was not achieved in patients with oncologic disease due to chemotherapy.

Infection was the second most common type of failure (13 cases, 15.9%). Two patients were managed by debridement only and retained their prosthesis, while three patients did a single-stage revision. In the other eight patients, the prosthesis was removed and replaced by an antibiotic spacer; 4 of the 8 patients lived with the spacer and refused to do any other surgeries, while two patients did a two-stage revision and one patient ended with fusion by a vascularized fibula. All the infected cases at the end of the study were free of infection except one. He had an amputation after 4 years due to persistent infection. In total, 23.1% of infected patients ended with poor MSTS score (15 or less) and 30.8% suffered from flexion deformity. Therefore, infection was found to markedly affect the final range of motion as well as the MSTS score. Infection is a major complication and its incidence varies in the literature from 3% to 20%.^15,21,30,31^ None of the factors related to the patient or the operation was found to significantly affect the rate of infection.

Structural failure in our study, including periprosthetic fractures and broken prosthesis, occurred in 15 patients (18.3%): 9 (11%) had periprosthetic fractures and only one case needed revision, while all the others were managed by casting or open reduction and internal fixation. Periprosthetic fracture did not affect prosthesis preservation rates. Six patients (7.3%) broke their prostheses and all were managed by revision. The incidence of broken prostheses was not significantly affected by the type of the prosthesis and whether it was cemented or not. Bus et al.^20^ found the incidence of broken prostheses to be 10%, while Zhang et al.^8^ found the incidence to be 5.6%. His average follow-up period was 53.2 months (range, 12–125 months), which is less than our follow-up period.

Finally, the last aim of this study was to evaluate the rates of prosthesis preservation and limb salvage. A total of 47 patients (57.3%) did not develop any form of complication, while 35 patients developed one or more complications. Among those who developed complications, 31 needed removal or revision of the prosthesis. By the end of the study, 51 (62.2%) patients had retained their implants. The estimated overall 5- and 10-year implant survival rates were 67.7% and 52.4%, respectively. Many studies found the 5-year prosthesis preservation rates to range from 57% to 83% and the 8- or 10-year rates to range from 45% to 77%.^9–11,24,32^

To our knowledge, we did not find any study that specifically evaluated distal femoral modular prosthesis in management of distal femoral osteosarcoma. This resulted in making comparisons between different studies not always applicable, as most of these studies used different types and techniques of endoprosthesis as well as different diagnoses, not just osteosarcoma. In addition, some of the studies excluded the local recurrence in their prosthesis survivorship rates such as Pala et al.^9^ and Capanna et al.^17^ They claimed that local recurrence was something not related to the implant, but rather to the aggressiveness and method of resection of the tumor.

Prosthesis preservation in our study was affected by infection, aseptic loosening, and broken prosthesis as well as cemented prosthesis. Capanna et al. found that cemented and cementless prosthesis did not affect prosthesis preservation,^17^ while Zhang et al. thought that cemented implants with a rotating hinge provided stable fixation, reduced the risk of loosening and bushing wear, and improved prosthesis survivorship.^8^

The incidence of amputation was 2.4% (two patients); one due to local recurrence and another due to persistent infection. Our 5- and 10-year rates of limb salvage were 98.8%. The rate of amputation in literature following limb salvage in the distal femur ranged from 2.5% to 25%.^8–10,18,32^

This study has some limitations as it is a non-randomized case series that is open to selection bias, but it has the advantage of analyzing many outcomes. This study spanned over two decades, so the use of chemotherapy and prosthetic designs changed over time. The learning curve and the technical experience has changed over the two decades, and this is evidenced by the fact that the incidence of infection and aseptic loosening was higher earlier in the study. Some of the patients included in this study were followed up for 1 year only, as they underwent operations recently, so they may need longer follow-up period to accurately detect the outcome and development of any failures.

## Conclusions

This study showed that patients with osteosarcoma distal femur who are treated by chemotherapy and limb salvage have an excellent long-term prognosis in terms of patient as well as limb survivorship. The use of modular endoprosthesis in these patients offered an acceptable function, with two-thirds of the patients retaining their prosthesis after 5 years and more than half retaining them after 10 years. Associated complications are manageable and do not affect limb survivorship.

## References

[1] Cho WH, Song WS, Jeon DG, Kong CB (2010). Differential presentations, clinical courses, and survivals of osteosarcomas of the proximal humerus over other extremity locations. Ann Surg Oncol.

[2] Kaste Sue C, Liu T, Billups CA, Daw NC (2004). Tumor size as a predictor of outcome in pediatric non-metastatic osteosarcoma of the extremity. Pediatr Blood Cancer.

[3] Fletcher CDM, Bridge JA, Hogendoorn P et al. WHO Classification of Tumours of Soft Tissue and Bone. 4th ed. World Health Organization, *WHO Press*, Geneva, 2013;5(5).

[4] Ferrari S, Serra M (2015). An update on chemotherapy for osteosarcoma. Exp Opin Pharmacother..

[5] Henderson ER, O’Connor MI, Ruggieri P, Windhager R (2014). Classification of failure of limb salvage after reconstructive surgery for bone tumours? A modified system including biological and expandable reconstructions. Bone Jt J.

[6] Chan YH (2003). Biostatistics 103: Qualitative data –tests of independence. Singapore Med J.

[7] Chan YH (2004). Biostatistics 203: Survival analysis. Singapore Med J.

[8] Zhang Chunlin, Jianping Hu, Zhu Kunpeng, Cai Tao (2018). Survival, complications and functional outcomes of cemented megaprostheses for high-grade osteosarcoma around the knee. Int Orthop.

[9] Pala E, Trovarelli G, Calabro T, Angelini A (2015). Survival of modern knee tumor Megaprostheses: failures, functional results, and a comparative statistical analysis. Clin Orthop Relat Res.

[10] Mattei JC, Chapat B, Ferembach B, Le Nail LR et al (2020); Fixed-hinge cemented modular implants: an effective reconstruction technique following primary distal femoral Bone tumor resection. A 136-case multicentre series. *Orthop Traumatol Surg Res*. 10.1016/j.otsr.2019.10.029 32205080

[11] Gosheger G, Gebert C, Ahrens H, Streitbuerger A (2006). Endoprosthetic reconstruction in 250 patients with sarcoma. Clin Orthop Relat Res.

[12] Ahlmann ER, Menendez LR, Kermani C, Gotha H (2006). Survivorship and clinical outcome of modular endoprosthetic reconstruction for neoplastic disease of the lower limb. J Bone Joint Surg Br..

[13] Frink SJ, Rutledge J, Lewis VO, Lin PP (2005). Favourable long-term results of prosthetic arthroplasty of the knee for distal femur neoplasms. Clin Orthop Relat Res.

[14] Sharil A, Nawaz A, Nor Azman M (2013). Early functional outcome of resection and endoprosthesis replacement for primary tumor around the knee. Malays Orthop J..

[15] Houdek MT, Wagner ER, Wilke BK (2016). Long term outcomes of cemented endoprosthetic reconstruction for periarticular tumours of the distal femur. Knee.

[16] Schwartz AJ, Kabo JM, Eilber FC, Eilber FR (2010). Cemented distal femoral endoprostheses for musculoskeletal tumour improved survival of modular versus custom implants. Clin Orthop Relat Res.

[17] Capanna R, Scoccianti G, Frenos F (2015). What was the survival of megaprostheses in lower limb reconstructions after tumor resections?. Clin Orthop Relat Res.

[18] Mazaleyrat M, Le Nail LR, Auberger G, David Biau et al. Survival and complications in hinged knee reconstruction prostheses after distal femoral or proximal tibial tumor resection: a retrospective study of 161 cases. *Orthop Traumatol Surg Res*. 2020;106(3):206-211 10.1016/j.otsr.2019.11.027 32276844

[19] Tan PX, Yong BC, Wang J (2012). Analysis of the efficacy and prognosis of limb salvage surgery for osteosarcoma around the knee. EJSO.

[20] Bus MPA, van de Sande MAJ, Fiocco M, Schaap GR (2017). What are the long-term results of MUTARS (a(R)) modular endoprostheses for reconstruction of tumor resection of the distal femur and proximal tibia?. Clin Orthop Relat Res.

[21] Orlic D, Smerdelj M, Kolundzic R, Bergovec M (2006). Lower limb salvage surgery: modular endoprosthesis in bone tumour treatment. Int Orthop.

[22] Bacci G, Ferrari S, Bertoni F, Ruggieri P (2000). Long term outcome for patients with non-metastatic osteosarcoma of the extremity treated at the Istituto Ortopedico Rizzoli according to the Istituto Ortopedico Rizzoli/osteosarcoma-2 protocol: an updated report. J Clin Oncol..

[23] Zeegen EN, Aponte-Tinao LA, Hornicek FJ (2004). Survivor analysis of 141 modular metallic endoprostheses at early follow up. Clin Orthop Relat Res..

[24] Myers GJC, Abudu AT, Carter SR, Tillman RM et al. The long-term results of endoprosthetic replacement of the proximal tibia for bone tumours. *J Bone Joint Surg* 2007;(Am Vol) 89B (12): 1632–1637. 10.1302/0301-620X.89B12.19481 18057365

[25] Batta V, Coathup MJ, Parratt MT, Pollock RC (2014). Uncemented, custom-made, hydroxyapatite-coated collared distal femoral endoprostheses. Bone Joint J.

[26] Hardes J, Henrichs MP, Gosheger G (2013). Endoprosthetic replacement after extra-articular resection of bone and soft-tissue tumours around the knee. Bone Joint J.

[27] Morgan HD, Cizik AM, Leopold SS (2006). Survival of tumor megaprostheses replacements about the knee. Clin Orthop Relat Res.

[28] Staats Kevin, Vertesich Klemens, Sigmund Irene K (2020). Does a competing risk analysis show differences in the cumulative incidence of revision surgery between patients with oncologic and non-oncologic conditions after distal femur replacement. Clin Orthop Relat Res.

[29] Pugh LR, Clarkson PW, Phillips AE, Biau DJ, Masri BA (2014). Tumor endoprosthesis revision rates increase with peri-operative chemotherapy but are reduced with the use of cemented implant fixation. J Arthroplasty..

[30] Cabas-Geat A.E, Bruchmann M.G, Albergo J.I et al. Modular prosthesis reconstruction after tumour resection, evaluation of failures and survival. *Rev Esp Cir Ortop Traumatol*. 2019;63(3):173-180 10.1016/j.recot.2019.01.003 30922597

[31] Bhangu AA, Kramer MJ, Grimer RJ, O’Donnell RJ (2006). Early distal femoral endoprosthetic survival: cemented stems versus the compress implant. Int Orthop..

[32] Niimi R, Matsumine A, Hamaguchi T (2012). Prosthetic limb salvage surgery for bone and soft tissue tumours around the knee. Oncol Rep.

